# World Health Organization fracture risk assessment tool in the assessment of fractures after falls in hospital

**DOI:** 10.1186/1472-6963-10-106

**Published:** 2010-04-27

**Authors:** Shin-ichi Toyabe

**Affiliations:** 1 Niigata University Crisis Mangement Office, Niigata University Hospital, Asahimachi-dori 1-754, Chuoku, Niigata City 951-8520, Japan

## Abstract

**Background:**

Falls are very common accidents in a hospital. Various risk factors and risk assessment tools are used to predict falls. However, outcomes of falls such as bone fractures have not been considered in these risk assessment tools, and the performance of risk assessment tools in a Japanese hospital setting is not clear.

**Methods:**

This was a retrospective single-institution study of 20,320 inpatients aged from 40 to 90 years who were admitted to a tertiary-care university hospital during the period from April 2006 to March 2009. Possible risk factors for falls and fractures including STRATIFY score and FRAX™ score and information on falls and their outcome were obtained from the hospital information system. The datasets were divided randomly into a development dataset and a test dataset. The chi-square test, logistic regression analysis and survival analysis were used to identify risk factors for falls and fractures after falls.

**Results:**

Fallers accounted for 3.1% of the patients in the development dataset and 3.5% of the patients in the test dataset, and 2.6% and 2.9% of the fallers in those datasets suffered peripheral fractures. Sensitivity and specificity of the STRATIFY score to predict falls were not optimal. Most of the known risk factors for falls had no power to predict fractures after falls. Multiple logistic analysis and multivariate Cox's regression analysis with time-dependent covariates revealed that FRAX™ score was significantly associated with fractures after falls.

**Conclusions:**

Risk assessment tools for falls are not appropriate for predicting fractures after falls. FRAX™ might be a useful tool for that purpose. The performance of STRATIFY to predict falls in a Japanese hospital setting was similar to that in previous studies.

## Background

Falls are very common accidents in a hospital [1]. Falls in a hospital often cause severe injuries such as bone fractures, soft tissue injuries and hematomas. About 3-10% of falls in hospitals result in physical injuries including fractures [2]. Risk of hip fracture has been shown to be eleven-times greater in hospital patients than in the general community [3]. These injuries may lead to additional healthcare costs, prolonged length of hospital stay and psychological distress for the patients. This situation might result in complaints and litigation from families of the patients [4].

A strategy that has been shown to be successful in preventing falls of inpatients is a target prevention strategy by selecting patients at high risk for falls [5-7]. Several clinical characteristics have been shown to be associated with increased incidence of falls in a hospital, and various risk assessment tools for falls have been developed [4,8-13]. In Japan, even the performance of popular risk assessment tools for inpatient falls has not been examined, making international comparison difficult [1,12]. A more important problem of these risk assessment tools is that these tools were developed to find patients at high risk for falls and not to predict patients who would suffer physical injuries after falls. In reality, more than 90% of inpatient falls do not result in physical injuries [2], but the costs attributable to falls are highly skewed to those that result in physical injuries. One of the most important reasons for preventing falls should be to prevent fractures and other severe injuries [5]. Risk assessment tools are needed to predict falls that are likely to be complicated with severe injuries such as fractures.

Measurement of bone mineral density (BMD) is the standard tool to assess susceptibility to fracture, but it is costly and impractical to measure BMD in all inpatients. Recently, fractures risk assessment tool (FRAX™) was developed by the World Health Organization [14-16]. FRAX™ has the advantage that it can be used without information on BMD and is adjusted for ethnic differences. The aims of this study were (i) to analyze the risk factors for fractures after falls among various patient characteristics including FRAX™ score and (ii) to examine the performance of the STRATFY tool (St. Thomas risk assessment tool in falling elderly) [17] in a Japanese hospital setting.

## Methods

### Settings

This study was conducted at Niigata University Hospital, an 810-bed academic teaching hospital in the city of Niigata. There are 23 clinical departments and the service area of the hospital as a tertiary care hospital covers all districts in Niigata Prefecture, which has a population of 2,400,000. All patients who had been admitted to the hospital during the period from April 2006 to March 2009 and who were aged from 40 to 90 years at admission were studied. During that period, 20,973 patients were admitted to the hospital, but 653 patients were excluded from the study because of missing data. Finally, data were obtained for a total of 20,320 patients aged from 40 to 90 years (median, 65.0 years; 25th percentile, 56.0 years; 75th percentile, 74.0 years). The patients included 9,738 females and 10,582 males, and 4,949 (24.4%) of the patients required acute admission. The dataset was randomly divided into two datasets of the same sizes by a person blinded to our study. One dataset was used for receiver operating curve (ROC) analysis to determine cut-off values (development dataset) and the other was used for validation of the analysis (test dataset).

### Risk assessment tools for falls and fractures

Various risk assessment tools for prediction of inpatient falls have been developed, but only the STRATIFY tool and the Morse Falls Scale [18] have been subjected to prospective validation in several cohorts with appropriate tests of predictive validity [7,8]. The STRATIFY tool showed high sensitivity and specificity in the original study, and its simplicity has facilitated its wide use in clinical practice [19]. We therefore used this tool in our study to assess patients' risk for falls. However, systematic review of the STRATIFY tool revealed that the tool may not be optimal for identifying individuals at high risk for falls and that population and setting affect performance of the tool [10]. The risk factors of fractures related to osteoporosis are age, prior fragility fracture, parental history of hip fracture, smoking, use of systemic corticosteroids, excess alcohol intake and rheumatoid arthritis [15]. By integrating these risk factors, WHO proposed the FRAX™ tool to compute ten-year probability of osteoporotic fracture. The FRAX™ tool has the advantage that it can be used without information on BMD and is adjusted for ethnic differences. Actually, it is used to determine thresholds for therapeutic intervention in a Japanese setting [14]. We therefore used the tool in this study to assess the risk for fracture.

### Data collection

Information on patients' background such as age, gender, body weight, height, history of bone fractures, smoking history, alcohol consumption, prescription of drugs, coexisting illness, admission day and discharge day was obtained from the hospital information system. Information on risk factors for falls was obtained from medical charts of the patients and fall assessment records completed by attending nurses at admission [8]. They included history of falls, gait instability, agitated confusion, urinary incontinence or frequency, visual impairment, lower limb weakness and prescription of 'culprit' drugs. The assessment was performed again when fall events occurred. Therefore, STRATIFY score [17] and FRAX™ score [14,16] were calculated at admission and when the fall events occurred. STRATIFY score was calculated on the basis of the original method [17], and FRAX™ score was based on the ten-year probability of major osteoporotic fracture according to body mass index.

### Falls and fractures after falls

The clinical outcome we studied was fallers with or without fractures rather than falls [9,20]. Data on fall events were obtained from online incident reports, records of x-ray order entry and medical charts of the patients. The incident reports concerning fall events were documented by the attending nurse and other medical staff, and the reports contained data on time, location, injury sustained and potential causative factor for falls. Peripheral fractures verified by radiological findings were included, but vertebral fractures were excluded from the study [20].

### Statistical analysis

Fall events and fracture after falls were analyzed by two different methods. The first method is the traditional chi-square test and multiple logistic analysis, which has been used for analysis of inpatient falls. For multiple logistic analysis, significant risk factors were selected by using the stepwise selection method and by the forced entery method. The second method is survival analysis in which time between admission and the event (falls or fractures after falls) during the hospital stay was considered as survival time. Discharge of the patient without fall events was considered as censoring. The Kaplan-Meier method was used for the analysis, and the logrank test was used to examine whether each risk factor was significantly associated with events. Multivariate Cox's proportional hazards regression model with time-dependent covariates was used to examine the risk factors that were most significantly associated with events among the various risk factors. Since values of several risk factors such as history of falls varied over time, these factors were included in the model by defining them as time-dependent covariates. Significant factors were selected by the stepwise selection method and by the forced entry method. A cut-off value to distinguish patients at risk from patients not at risk was determined on the basis of results of ROC analysis of data in the development dataset. The value corresponding to the nearest point of the ROC curve to the top left-hand corner was chosen as a cut-off value to distinguish patients at risk from patients not at risk [21]. Sensitivity, specificity and area under the ROC curve (AUC) were calculated. Distribution of continuous data was shown by medians (25-percentiles, 75-percentiles). All statistical analyses were performed using SPSS Statistics 17.0 (SPSS Japan Inc., Tokyo, Japan), and a p-value less than 0.05 was considered significant.

### Ethical consideration

All data were analyzed anonymously. The Ethical Committee of Niigata University School of Medicine gave ethical approval.

## Results

### Falls and fractures after falls

The numbers of patients who experienced more than one fall during admission were 308 (3.1%) in the development dataset and 345 (3.4%) in the test dataset. Eight (2.6%) of the fallers in the development dataset and 10 (2.9%) of the fallers in the test dataset suffered peripheral fractures after falls. The ages of the patients who suffered fractures after falls were 78.0 (64.0, 83.0) years for the development dataset and 75.0 (64.5, 76.5) years for the test dataset. All but one of the patients who suffered peripheral fractures broke bones during the first fall.

### Risk factors for falls

Univariate analyses (chi-square test and logrank test) revealed that various known risk factors including dichotomized STRATIFY score were significantly associated with fallers (Table 1). The cut-off value of the STRATIFY score to predict falls was determined to be a value of 2 based on results of ROC analysis of data in the development dataset. Proportions of high-risk patients based on the cut-off value of STRATIFY score were 26.5% in the development dataset and 26.8% in the test dataset. Sensitivity and specificity of the cut-off value to predict falls were 0.648 and 0.749 in the development dataset and 0.678 and 0.749 in the test dataset, respectively (Table 2). LOS was dichotomized at 14 days, which was the median value for all patients. Only the factor 'visual impairment' was not significant. When the risk factors as well as STRATIFY score were entered simultaneously into the multiple logisitc model, factors shown in Table 3A were significantly associated. On the other hand, multivariate Cox's regression analysis showed that the factors in Table 3B were significantly associated with falls. In both datasets, the factor 'history of falls', factor 'gait instability' and STRATIFY score were significantly associated with falls in both analyses.

**Table 1:** Results of univariate analysis of risk factors for falls.

Dataset	Items	Number of patients		Sig.	
			
		Fallers	Non fallers	Chi-square test	Logrank test
Development dataset (n = 10,160)	n	308	9,852		
	History of falls	115	677	<0.001	<0.001
	Gait instability	84	774	<0.001	<0.001
	Agitated confusion	85	513	<0.001	<0.001
	Urinary incontinence/frequency	65	785	<0.001	<0.001
	Visual impairment	69	1,807	0.083	0.154
	Lower limb weakness	143	1,614	<0.001	<0.001
	Prescription of 'culprit' drugs	79	1,158	<0.001	<0.001
	STRATIFY score ≥ 2	190	2,055	<0.001	<0.001
	LOS ≥ 14	157	4,836	0.552	-

Test dataset (n = 10,160)	n	345	9,815		
	History of falls	132	681	<0.001	<0.001
	Gait instability	100	735	<0.001	<0.001
	Agitated confusion	81	519	<0.001	<0.001
	Urinary incontinence/frequency	76	801	<0.001	<0.001
	Visual impairment	74	1,811	0.181	0.158
	Lower limb weakness	157	1,555	<0.001	<0.001
	Prescription of 'culprit' drugs	83	1,165	<0.001	0.002
	STRATIFY score ≥ 2	221	2,039	<0.001	<0.001
	LOS ≥ 14	193	4,917	0.038	-

Various risk factors for falls were evaluated to determine whether they are associated with falls by using the chi-square test and logrank test in development and test datasets. STRATIFY score and LOS were dichotomized at 2 and 14, respectively. LOS, length of hospital stay.

**Table 2:** Sensitivity and specificity of the STRATIFY score and the FRAX™ score.

A								
			
Items	Events	Datasets	AUC	95% CI				
							
				lower	upper			
			
STRATIFY	Falls	Development dataset	0.749	0.719	0.779			
		Test dataset	0.765	0.736	0.794			
				
	Fracture after falls	Development dataset	0.717	0.549	0.885			
		Test dataset	0.636	0.464	0.808			
			
FRAX	Falls	Development dataset	0.606	0.574	0.637			
		Test dataset	0.589	0.557	0.620			
				
	Fracture after falls	Development dataset	0.749	0.580	0.917			
			
		Test dataset	0.727	0.552	0.901			
			
B								
**Items**	**Events**	**Datasets**	**Sensitivity**	**95% CI**		**Specificity**	**95% CI**	
						
				**lower**	**upper**		**lower**	**upper**

STRATIFY	Falls	Development dataset	0.648	0.592	0.701	0.749	0.740	0.759
		Test dataset	0.678	0.625	0.726	0.749	0.739	0.758
	
	Fracture after falls	Development dataset	0.625	0.306	0.863	0.736	0.726	0.745
		Test dataset	0.400	0.168	0.687	0.732	0.723	0.742

FRAX	Falls	Development dataset	0.481	0.425	0.536	0.663	0.654	0.672
		Test dataset	0.496	0.443	0.548	0.666	0.657	0.675
	
	Fracture after falls	Development dataset	0.750	0.409	0.929	0.659	0.620	0.668
		Test dataset	0.700	0.397	0.892	0.661	0.652	0.670

AUC (A), sensitivity and specificity (B) of the STRATIFY score and the FRAX™ score to detect falls and fracture after falls were calculated. Cut-off values for the STRATIFY score and the FRAX™ score were set at 2 and 10, respectively.

**Table 3:** Results of multivariate analysis of risk factors for falls.

A						
Datasets	Items	Estimated coefficient (β)	Standard error for β	Sig.	OR	95% CI for OR
Development dataset	History of falls	0.919	0.169	<0.001	2.507	1.801 - 3.489
	Gait instability	0.451	0.153	0.003	1.571	1.164 - 2.119
	Agitated confusion	0.698	0.180	<0.001	2.009	1.412 - 2.859
	Lower limb weakness	0.444	0.143	0.002	1.559	1.178 - 2.063
	STRATIFY score	0.335	0.072	<0.001	1.397	1.214 - 1.609
	Constant	-4.341	0.110	<0.001	0.013	

Test dataset	History of falls	0.759	0.155	<0.001	2.137	1.577 - 2.897
	Gait instability	0.526	0.145	<0.001	1.692	1.275 - 2.247
	Lower limb weakness	0.332	0.137	0.015	1.394	1.066 - 1.824
	STRATIFY score	0.550	0.058	<0.001	1.734	1.547 - 1.944
	Constant	-4.420	0.109	<0.001	0.012	

**B**						

**Datasets**	**Items**	**Estimated coefficient (β)**	**Standard error for β**	**Sig.**	**HR**	**95% CI for HR**

Development dataset	History of falls	0.945	0.158	<0.001	2.572	1.888 - 3.505
	Gait instability	0.470	0.139	0.001	1.599	1.217 - 2.102
	Agitated confusion	0.475	0.167	0.005	1.609	1.158 - 2.233
	Lower limb weakness	0.273	0.136	0.045	1.313	1.006 - 1.714
	STRATIFY score	0.265	0.069	<0.001	1.303	1.139 - 1.492

Test dataset	History of falls	0.677	0.147	<0.001	1.968	1.475 - 2.627
	Gait instability	0.606	0.128	<0.001	1.834	1.428 - 2.354
	STRATIFY score	0.469	0.052	<0.001	1.598	1.442 - 1.770

Risk factors shown in Table 1 were analyzed by multiple logistic regression analysis (A) and multivariate Cox's regression analysis in development and test datasets (B). Significant factors were selected by using the stepwise selection method. CI, confidence interval; LOS, length of hospital stay; OR, odds ratio; HR, hazards ratio.

### Risk factors for fractures after falls

**Figure 1:**
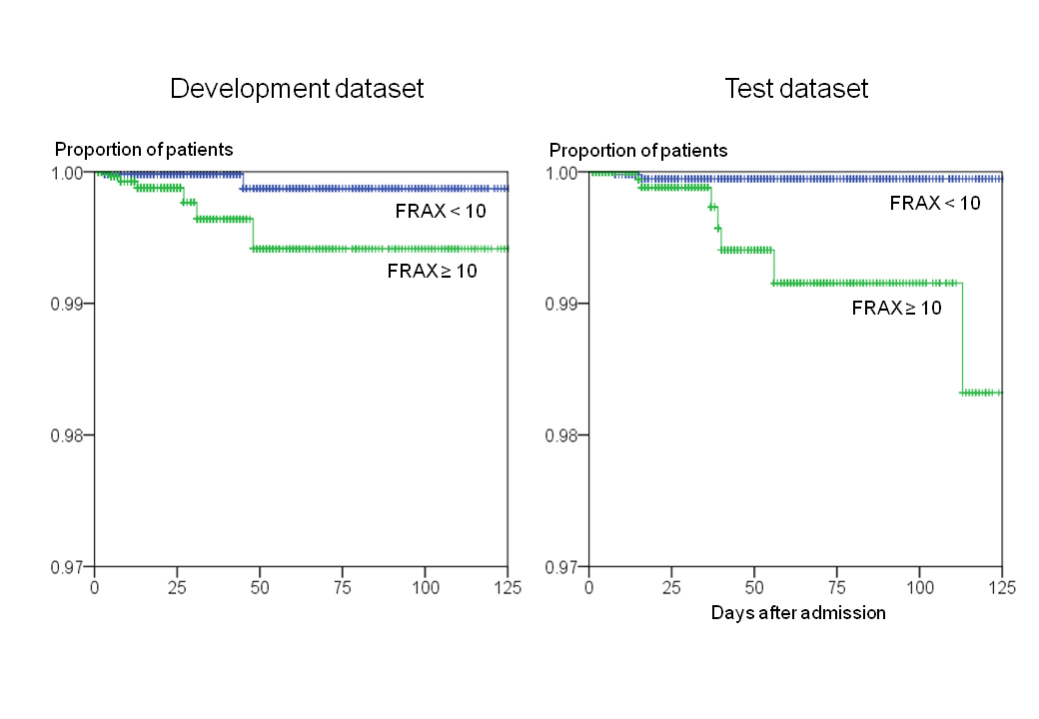
**Survival plots for fractures after falls**. All patients were plotted on the Kaplan-Meier survival curve as a function of length of stay. Cumulative rates of fractures after falls were compared between patients with FRAX™ score of more than 10 and patients with FRAX™ score of less than 10.

The chi-square test revealed that the factor 'lower limb weakness' and the FRAX™ score were significantly associated with fracture after falls in both datasets (Table 4). In addition, dichotomized STRATIFY score was a significant factor in the development dataset and dichotomized LOS was a significant factor in the test dataset. The cut-off value of the FRAX™ score to predict fracture after falls was determined to be as a value of 10 based on results of ROC analysis of data in the development dataset. Proportions of high-risk patients based on the cut-off value of FRAX™ score were 34.1% in the development dataset and 33.9% in the test dataset. Sensitivity and specificity of the cut-off value were 0.750 and 0.659 in the development dataset and 0.700 and 0.661 in the test dataset, respectively (Table 2B). On the other hand, the logrank test revealed that only dichotomized FRAX™ score was significantly associated with fractures after falls both in the development dataset and the test dataset (Table 4, Figure 1). When the risk factors shown in Table 4 were entered into the multiple logisitc regression model, only FRAX™ score was selected as a factor significantly associated with fractures after falls by the stepwise selection method both in the development dataset and the test dataset (Table 5A). Similarly, multivariate Cox's regression analysis showed that only FRAX™ score was a significant factor (Table 5B). Next, we analyzed how the combination of STRATIFY and FRAX™ tools would work for prediction of fracture after falls. Both scores were forcibly entered into the multiple logisitc model (Table 5C) and multivariate Cox's regression model (Table 5D), but only FRAX™ score was significantly associated with fracture after falls in Cox's regression analysis.

**Table 4:** Results of univariate analysis of risk factors for fractures after falls.

Dataset	Items	Number of patients		Sig.	
			
		Fallers with fracture after falls	Non fallers and fallers without fractures after falls	Chi-square test	Logrank test
Development dataset (n = 10,160)	n	8	10,152		
	History of falls	2	790	0.248	0.233
	Gait instability	0	858	0.823	0.292
	Agitated confusion	2	596	0.122	0.162
	Urinary incontinence/frequency	0	850	0.829	0.306
	Visual impairment	3	1,873	0.351	0.200
	Lower limb weakness	4	1,753	0.048	0.110
	Prescription of 'culprit' drugs	3	1,234	0.099	0.140
	STRATIFY score ≥ 2	5	2,240	0.020	0.071
	FRAX score ≥ 10.0	6	3,561	0.046	0.019
	LOS ≥ 14	4	4,989	1.000	-

Test dataset (n = 10,160)	n	10	10,150		
	History of falls	2	811	0.415	0.599
	Gait instability	1	835	1.000	0.777
	Agitated confusion	1	600	1.000	0.915
	Urinary incontinence/frequency	2	877	0.475	0.402
	Visual impairment	4	1,885	0.182	0.055
	Lower limb weakness	5	1,712	0.018	0.143
	Prescription of 'culprit' drugs	2	1,248	0.795	0.920
	STRATIFY score ≥ 2	4	2,260	0.334	0.819
	FRAX score ≥ 10.0	7	3,518	0.044	0.025
	LOS ≥ 14	9	5,110	0.028	-

Various risk factors for falls, STRATIFY score and FRAX™ score were evaluated to determine whether they are associated with fractures after falls by using the chi-square test and logrank test. LOS, length of hospital stay. FRAX™ score was dichotomized at 10.

**Table 5:** Results of multivariate analysis of risk factors for fractures after falls.

A						
**Datasets**	**Items**	**Estimated coefficient (β)**	**Standard error for β**	**Sig.**	**OR**	**95% CI for OR**

Development dataset	FRAX score	0.069	0.026	0.008	1.072	1.018 - 1.128
	Constant	-7.865	0.572	<0.001	0.000	

Test dataset	FRAX score	0.076	0.024	0.001	1.079	1.030 - 1.130
	Constant	-7.726	0.524	<0.001	0.000	

						

**B**						

**Datasets**	**Items**	**Estimated coefficient (β)**	**Standard error for β**	**Sig.**	**HR**	**95% CI for HR**

Development dataset	FRAX score	0.063	0.025	0.012	1.065	1.014 - 1.119

Test dataset	FRAX score	0.065	0.022	0.003	1.067	1.022 - 1.114

						

**C**						

**Datasets**	**Items**	**Estimated coefficient (β)**	**Standard error for β**	**Sig.**	**OR**	**95% CI for OR**

Development dataset	STRATIFY score	0.443	0.256	0.083	1.557	0.943 - 2.569
	FRAX score	0.060	0.028	0.034	1.062	1.004 - 1.122
	Constant	-8.358	0.697	<0.001	0.000	

Test dataset	STRATIFY score	0.289	0.241	0.231	1.335	0.832 - 2.141
	FRAX score	0.070	0.025	0.004	1.072	1.022 - 1.125
	Constant	-8.020	0.603	<0.001	0.000	

						

**D**						

**Datasets**	**Items**	**Estimated coefficient (β)**	**Standard error for β**	**Sig.**	**HR**	**95% CI for HR**

Development dataset	STRATIFY score	0.330	0.257	0.200	1.391	0.840 - 2.302
	FRAX score	0.056	0.027	0.035	1.058	1.004 - 1.115

Test dataset	STRATIFY score	0.153	0.240	0.523	1.165	0.729 - 1.864
	FRAX score	0.064	0.022	0.004	1.066	1.020 - 1.114

Various risk factors for falls, STRATIFY score and FRAX™ score were evaluated in development and test datasets to determine whether they are associated with fracture after falls by using multiple logistic regression analysis (A) and multivariate Cox's regression analysis (B). Significant factors were selected by using the stepwise selection method. In Tables C and D, both STRATIFY score and FRAX™ score were forcibly entered into the logistic model (C) and Cox's regression model (D). CI, confident interval; LOS, length of hospital stay; OR, odds ratio; HR, hazards ratio.

### Risk factors in aged patients

The performance of the two risk tools in a much more selected population was evaluated. We performed multiple logisitc analysis and multivariate Cox's regression analysis by entering the risk factors as well as the two risk scores simultaneously into the models for patients more than 65 years old, who were at higher risk for both falls and fractures. The results were almost the same as the results of analysis for all subjects (Table 6). The factor 'history of falls', the factor 'gait instability' and STRATIFY score were significantly associated with falls both in multiple logistic regression analysis (Table 6A) and multivariate Cox's regression analysis (Table 6B). FRAX™ was significantly associated with fracture after falls in the development dataset analyzed by multivariate Cox's regression analysis and in the test dataset analyzed by both methods.

**Table 6:** Risk factors for falls and for fractures after falls in patients over 65 years old.

A						
**Datasets**	**Items**	**Estimated coefficient (β)**	**Standard error for β**	**Sig.**	**OR**	**95% CI for OR**

Development dataset	History of falls	0.971	0.199	<0.001	2.640	1.788 - 3.898
	Gait instability	0.527	0.182	0.004	1.694	1.185 - 2.422
	Agitated confusion	0.731	0.212	0.001	2.078	1.373 - 3.145
	Lower limb weakness	0.397	0.171	0.020	1.487	1.063 - 2.079
	STRATIFY score	0.275	0.086	0.001	1.316	1.112 - 1.558
	Constant	-4.205	0.140	<0.001	0.015	

Test dataset	History of falls	0.727	0.183	<0.001	2.069	1.447 - 2.959
	Gait instability	0.574	0.172	0.001	1.775	1.268 - 2.484
	Lower limb weakness	0.346	0.160	0.031	1.413	1.033 - 1.933
	STRATIFY score	0.459	0.069	<0.001	1.583	1.382 - 1.814
	Constant	-4.142	0.132	<0.001	0.016	

						

**B**						

**Datasets**	**Items**	**Estimated coefficient (β)**	**Standard error for β**	**Sig.**	**HR**	**95% CI for HR**

Development dataset	History of falls	0.977	0.188	<0.001	2.655	1.838 - 3.836
	Gait instability	0.576	0.163	<0.001	1.780	1.292 - 2.451
	Agitated confusion	0.534	0.198	0.007	1.705	1.156 - 2.514
	STRATIFY score	0.239	0.080	0.003	1.270	1.085 - 1.486

Test dataset	History of falls	0.594	0.172	0.001	1.812	1.294 - 2.536
	Gait instability	0.649	0.151	<0.001	1.913	1.424 - 2.571
	STRATIFY score	0.403	0.062	<0.001	1.497	1.325 - 1.690

						

**C**						

**Datasets**	**Items**	**Estimated coefficient (β)**	**Standard error for β**	**Sig.**	**OR**	**95% CI for OR**

Development dataset	Lower limb weakness	1.728	0.867	0.046	5.631	1.030 - 30.780
	Constant	-7.496	0.707	<0.001	0.001	

Test dataset	FRAX score	0.069	0.028	0.012	1.072	1.015 - 1.131
	Constant	-7.528	0.665	<0.001	0.001	

						

**D**						
**Datasets**	**Items**	**Estimated coefficient (β)**	**Standard error for β**	**Sig.**	**HR**	**95% CI for HR**

Development dataset	FRAX score	0.060	0.031	0.050	1.062	1.000 - 1.128

Test dataset	FRAX score	0.061	0.027	0.023	1.063	1.008 - 1.120

Various risk factors for falls, STRATIFY score and FRAX™ score were evaluated in development and test datasets to determine whether they are associated with falls (A, B) or fractures after falls (C, D) by using multiple logistic regression analysis (A, C) and multivariate Cox's regression analysis (B, D). Significant factors were selected by using the stepwise selection method. CI, confidence interval; LOS, length of hospital stay; OR, odds ratio; HR, hazards ratio.

## Discussion

In this study, we analyzed the risk factors for falls resulting in fractures, focusing on FRAX™ fracture risk assessment tool. Most of the risk factors for falls had no power to predict fracture after falls, but a high FRAX™ score was closely associated with fractures. This result was confirmed not only by conventional multiple logisitc analysis but also by survival analysis referring to the fracture after a fall as event and the time between admission and the event as survival time. By using the risk assessment tools for fractures, we could identify and target fallers that were at high risk for fractures more efficiently.

Our results suggest that a strategy to prevent fractures after falls might require programs for patients with bone fragilities apart from a program to prevent falls. The majority of patients who fell did not injure themselves and only 2.6% - 2.9% of patients who fell incurred a peripheral fracture. The risk assessment tools should be used for prediction of physical injuries resulting from falls, not for prediction of falls themselves. However, the existing risk assessment tools have poor performance in predicting not only fractures after falls but also falls themselves [10]. Several reports suggested that the risk assessment tools for falls have the same poor performance as clinical judgment of nurses [22-25]. The use of risk assessment tools for falls might have no clinical benefit and waste scarce nursing resources [23]. Hospitals should shift their emphasis from simply targeting high-risk patients for falls to identifying high-risk patients for fractures after falls and employing programs to prevent fractures. A targeted intervention for high-risk patients such as the use of a hip protector [26,27] might be beneficial even though the compliance with a hip protector remains to be improved [28]. Our results suggest that a FRAX™ score of more than 10 is useful for identifying the high-risk patients in terms of sensitivity. This cut-off point was consistent with the recommended threshold for therapeutic intervention for osteoporotic fracture in the Japanese population [14]. However, positive predictive value of FRAX™ score to predict fracture after falls was 0.003 in both datasets. The low positive predictive value was problematic when FRAX™ score was used as a means of screening [29]. More restrictive selection of patients at high risk for fractures is necessary.

Our study is the first study in which the performance of STRATIFY tools was examined in a Japanese setting, but it was found that the performance of the STRATIFY risk assessment tool is not optimal in a Japanese acute-care hospital setting. This tool was originally developed and validated in the United Kingdom to predict falls occurring in a hospital, and sensitivity and specificity were both in excess 80% in the original study [17]. However, systemic review of this tool has shown that this tool has poorer predictive accuracy than the results of the original study and that population and setting affect the performance of this tool [10]. Our results showed that the performance of the STRATIFY tool was not necessarily satisfactory in a Japanese hospital setting but was similar to that in previous studies [30]. Since a weighted risk score based on the STRATIFY tool could improve the performance of the tool [9], modification of the tool might be needed for a Japanese setting.

There are several limitations of this study. First, we limited the study subjects to patients aged from 40 to 90 years. This was because the FRAX™ scoring system targets that age group. Second, no validation study using a prospective cohort was performed in this study. A prospective study is needed to validate the usefulness of the assessment tool for predicting fractures after falls and to generalize the findings of our study. Third, sensitivity and specificity of the STRATIFY tool for falls, which were calculated by the standard approach, might be incorrect because falls might be recurrent and time-dependent [31]. Finally and most importantly, the sample size of our study was small in terms of number of fractures to construct proper statistical models. Further investigation using larger sample sizes is needed to determine whether fracture risk assessment is useful for predicting fractures after inpatient falls.

## Conclusions

Risk assessment tools for falls are not appropriate for predicting fractures after falls. FRAX™ might be a useful tool for that purpose. The performance of STRATIFY to predict falls in a Japanese hospital setting was similar to that in previous studies.

## Competing interests

The author declares that they have no competing interests.

## Authors' contributions

ST is solely responsible for this manuscript.

## Pre-publication history

The pre-publication history for this paper can be accessed here:

http://www.biomedcentral.com/1472-6963/10/106/prepub
